# Experiences of Pulmonary Rehabilitation in People Living with Chronic Obstructive Pulmonary Disease and Frailty. A Qualitative Interview Study

**DOI:** 10.1513/AnnalsATS.201910-800OC

**Published:** 2020-10

**Authors:** Lisa Jane Brighton, Katherine Bristowe, Joanne Bayly, Margaret Ogden, Morag Farquhar, Catherine J. Evans, William D. C. Man, Matthew Maddocks

**Affiliations:** ^1^ Cicely Saunders Institute of Palliative Care, Policy and Rehabilitation, and; ^2^ Cicely Saunders Institute Patient and Public Involvement Group, King’s College London, London, United Kingdom; ^3^ School of Health Sciences, University of East Anglia, Norwich, United Kingdom; ^4^ Sussex Community NHS Foundation Trust, Brighton General Hospital, Brighton, United Kingdom; ^5^ National Heart and Lung Institute, Imperial College, London, United Kingdom; and; ^6^ Royal Brompton and Harefield NHS Foundation Trust, Harefield Pulmonary Rehabilitation and Muscle Research Laboratory, London, United Kingdom

**Keywords:** chronic obstructive pulmonary disease, frailty, rehabilitation, exercise, qualitative research

## Abstract

**Rationale:** People living with both chronic obstructive pulmonary disease (COPD) and frailty have high potential to benefit from pulmonary rehabilitation but face challenges completing programs. However, research to understand ways to optimize participation in this group is lacking.

**Objectives:** To explore the experiences, needs, and preferences of people with COPD and frailty referred for outpatient pulmonary rehabilitation.

**Methods:** Semistructured interviews with people with COPD and physical frailty, purposively sampled by age, living status, level of frailty, and completion of pulmonary rehabilitation. Thematic analysis with a critical realist perspective was used, involving relevant stakeholders with clinical, academic, and lived experience for interpretive rigor.

**Results:** Nineteen people with COPD and frailty were interviewed, with a median age of 78 years (range, 58–88). Nine did not complete their pulmonary rehabilitation program. Four themes were identified: striving to adapt to multidimensional loss, tensions of balancing support with independence, pulmonary rehabilitation as a challenge worth facing, and overcoming unpredictable disruptions to participation. Participants described constantly adapting to their changing health and resulting multidimensional losses (e.g., functional abilities, relationships, confidence). This involved traversing between independence and seeking support, set against a mismatch between their needs and what support is available. People with COPD and frailty can be highly motivated to participate in pulmonary rehabilitation, despite the physical and mental demands it entails, and report a range of benefits. Yet in the context of changeable health, they must often overcome multiple unpredictable disruptions to completing rehabilitation programs. Participant determination and flexibility of services can facilitate ongoing attendance, but for some, these unpredictable disruptions erode their motivation to attend.

**Conclusions:** People with COPD and frailty experience accumulating, multidimensional loss. This group are motivated to complete pulmonary rehabilitation but often require additional support and flexibility owing to fluctuating and unpredictable health. Person-centered approaches should be considered to minimize disruptive health events and support pulmonary rehabilitation participation and completion. Service adaptations could allow more flexibility to meet the changing needs of this group and enable communication around how pulmonary rehabilitation might align with their priorities.

Chronic obstructive pulmonary disease (COPD) affects multiple body systems and has been described as reflecting an “accelerated aging” (1). COPD frequently occurs in the context of multimorbidity: more than 60% of people with COPD live with two or more additional health conditions (2). Related to this, people with COPD have twice the odds of living with frailty than people of a similar age without COPD (3).

Frailty is a multidimensional syndrome characterized by decreased reserve and diminished resistance to stressors (4). Physical dimensions of frailty are characterized by diminished strength and endurance and reduced physiological function (5). Recognition of frailty offers advantages over measures of disease severity, particularly in the context of multimorbidity, in that it incorporates a more holistic understanding of a person’s health and limitations (6). Pooled prevalence estimates suggest that 19% of people with COPD are living with frailty, whereas a further 56% are prefrail (3). People with COPD and frailty are at increased risk of mortality (7, 8) and readmission after hospitalization for an exacerbation of their disease (9). In comparison with their nonfrail counterparts, people with COPD and frailty experience poorer physical function and health status (10) as well as increased anxiety and depression symptoms (11) and are less likely to receive disease-modifying interventions (12).

Participating in exercise improves outcomes for people with COPD (13, 14) or frailty (15, 16) and is recommended by clinical guidelines for each condition (17, 18). For people with both COPD and frailty, pulmonary rehabilitation is associated with improvements in frailty status (11, 19), breathlessness, exercise performance, physical activity levels, and health status (11, 20). However, people with COPD and frailty are less likely to start, and complete, pulmonary rehabilitation (11).

People with COPD report multiple challenges to participation in exercise-based interventions, including lack of perceived benefit, concurrent burden of comorbid conditions, conflicts with other priorities, difficulties with mobility and travel, fear of worsening symptoms, low energy and motivation, and exacerbations of their COPD (21–23). Similar barriers are noted by people living with frailty, including conflicting commitments (e.g., hobbies, caring responsibilities), physical limitations (e.g., pain, fatigue), and challenges around access and travel (24, 25). Although some view exercise positively (24), others report disengaging owing to perceiving frailty as inevitable in older age, and feeling disempowered or depersonalized in their interactions with services (26).

Understanding (non-)participation and identifying optimal ways of supporting people with COPD and frailty is a priority for improving outcomes for this population (27). People with both COPD and frailty have high potential to gain from, but also a high likelihood of facing challenges to completing, pulmonary rehabilitation (11). Yet, research with people with COPD and frailty to understand their specific needs and challenges is lacking, and optimal models of exercise for this group are not well understood. We aimed to explore the experiences, needs and preferences of people living with both COPD and frailty referred for pulmonary rehabilitation, to optimize service delivery for this group. Our objectives were to: *1*) understand the experiences and preferences of people living with COPD and frailty; *2*) identify current support and areas of unmet need; and *3*) explore motivation for, and barriers to, continued participation in pulmonary rehabilitation.

## Methods

### Design

We conducted a qualitative interview study within a critical realist paradigm (28). This means participants’ responses were deemed to reflect a reality that can be understood through empirical means. Yet, we also acknowledge the influence of social and cultural structures in understanding this reality. We drew on theories around successful aging (29), self-regulation (30), and stress and coping (31) to develop a comprehensive topic guide and inform data interpretation. For example, successful aging theory (29) aided exploration of how people adapt, reprioritize, and compensate in response to losses in function in older age; the common-sense model of self-regulation (30) provided a framework for understanding interactions with services and broader health behaviors, and the transactional model of emotions and coping (31) supported our understanding of how coping arises from perceptions of stressors and available resources. Although we drew on specific theories with the intention of developing a richer explanation of reality, we were cognizant that they could be challenged by new data (28).

### Setting and Recruitment

We recruited participants from two London hospitals providing outpatient pulmonary rehabilitation. Clinical staff identified potential participants during their initial assessments for pulmonary rehabilitation. A researcher then periodically followed up with those interested in participating, so that they could be potentially sampled when they stopped or completed their pulmonary rehabilitation.

### Participants and Sampling

People referred for pulmonary rehabilitation with a physician diagnosis of COPD, who at initial assessment were identified as physically frail using the Short Physical Performance Battery (32) (SPPB; score of ≤9), were invited to participate. The SPPB scores performance across three tests: standing balance, habitual gait speed, and ability to stand. Total scores range from 0 (low function) to 12 (high function). Thresholds of ≤9 and ≤7 have been suggested to indicate prefrailty and frailty, respectively (33). Patients’ informal caregivers also participated if patients preferred. People under the age of 18 years, unable to speak English, or without capacity to provide informed consent were excluded.

We purposively sampled participants by age (> or ≤80 yr), living status (alone or with others), level of physical frailty (SPPB scores of > or ≤7), and completion of pulmonary rehabilitation (did or did not complete). Within the group who did not complete pulmonary rehabilitation, we attempted to sample those who were and were not admitted to hospital.

### Data Collection

A female researcher (L.B.) with a background in psychology and palliative care research (B.Sc., M.Sc.) conducted the interviews in participants’ preferred locations, between October 2018 and April 2019. L.B. had previous training in qualitative research and experience in conducting interviews with people with serious illness and their families. L.B. was not known to participants before the interviews.

The interviews followed a semistructured interview topic guide (online supplement E1) developed with input from people with lived experience relevant to both COPD and frailty, and their informal carers (service user representatives). The topic guide explored participants’ current health and priorities, support and unmet needs, and expectations and experiences of pulmonary rehabilitation. On the advice of the service user representatives, the researcher identified and used participants’ own language in relation to frailty, for example, slowing down, difficulties walking, lack of strength or energy. Service user representatives also prompted the researcher to consider the participant’s assets and resilience in addition to limitations. Interviews were audio-recorded and transcribed verbatim. The researcher completed detailed field notes to describe interview flow, contextual factors, participant responses, and initial reflections immediately after each interview.

Data collection continued until the data set was deemed to be approaching thematic saturation (34) (i.e., rich data with breadth and depth in relation to the study objectives, with evidence of replication across several participants [35]). To determine potential thematic saturation, we conducted a preliminary analysis of the detailed reflective field notes, considering the above definition while also reflecting on Malterud and colleagues’ (36) dimensions of information power. These dimensions consider the data in relation to the breadth of the study aim, sample specificity, level of existing contributing theory, dialogue quality, and the need for cross-case analysis.

### Analysis

We conducted a reflexive thematic analysis to identify patterns of meaning within the data (37). First, one researcher (L.B.) familiarized themselves with the data through revisiting the audio recordings, transcripts, and field notes. They generated initial codes to capture meaningful basic elements of the data in relation to the study objectives. A service user representative with qualitative analysis training (M.O.) also familiarized themselves with, and generated initial codes for, a sample of the data. Meanings were primarily considered at a semantic (explicit) level, but with consideration of latent (implicit) interpretations. L.B. inductively generated themes by reviewing and refining codes, and writing definitions accompanied by illustrative quotes. The themes and related codes were refined using three processes: revisiting the original interview data to ensure fair interpretation, comparing our findings with existing theory to assess if this may deepen our understanding, and review by stakeholders with differing backgrounds to work toward a richer and more nuanced understanding of the data (38). The latter included review by coauthors from different disciplines (e.g., nursing, physiotherapy) and representing relevant academic, clinical, and service user experiences. Finally, we constructed a narrative of the findings, with reference to illustrative quotes. Although described as a linear process, we moved forward and backward between the stages as thinking changed and progressed.

### Ethical Approval

The London Camberwell St Giles Research Ethics Committee (ref. 18/LO/1197) approved this study. We obtained written informed consent prior to interviews.

## Results

Of 49 eligible people introduced to the study, 19 were interviewed (Table 1). Sixteen people who were eligible and went on to complete their rehabilitation were not sampled after we reached saturation within this subgroup. Nine declined to be contacted, and five were lost to follow-up: two became too unwell, and three could not be contacted. Median interview length was 60 minutes (range, 30–120; interquartile range, 50–80); most took place in participants’ homes (*n* = 17), but two took place at the researcher’s university. In three interviews, participants were accompanied by a family member: two who consented for their contributions to be included and one who was present but did not participate.

**Table 1.** Qualitative interview participant characteristics (*n* = 19)

Characteristic	*N*/Median (Range)
Age, yr	78 (58–88)
GOLD spirometric stage*	
1 (mild)	1
2 (moderate)	3
3 (severe)	12
4 (very severe)	2
Physical frailty (SPPB) score at initial assessment	6 (1–9)
Long-term oxygen therapy	1
Number of comorbidities^†^	2 (0–5)
Sex	
F	10
M	9
Education	
Left school age 15 yr or younger	9
Left school age 16–19 yr	7
Postsecondary or university qualifications	3
Ethnicity	
Asian, Black, or Mixed	3
White British or Irish	16
Smoking history	
Current smoker	3
Ex-smoker	15
Never smoked	1
Sampling frame characteristics	
Aged over 80 yr	8
Physical frailty score <7	13
Living alone	11
Did not start or complete PR program^‡^	9

*Definition of abbreviations*: GOLD = Global Initiative for Chronic Obstructive Lung Disease; PR = pulmonary rehabilitation; SPPB = short physical performance battery.

* *n* = 1 missing from PR notes.

^†^ Most commonly reported comorbidities included arthritis, asthma, atrial fibrillation, and falls.

^‡^ *n* = 4 did not start, *n* = 5 did not complete.

Four themes were identified: striving to adapt to multidimensional loss, tensions of balancing support with independence, pulmonary rehabilitation as a challenge worth facing, and overcoming unpredictable disruptions to participation. Subthemes and illustrative quotes are shown in Tables 2–5.

**Table 2.** Striving to adapt to multidimensional loss: illustrative quotes

Subtheme	Illustrative Quote
Accumulation of health events and symptoms	*“Monday, when I went in there I said, ‘It’s actually the first time this year where, all it is, it’s just the COPD. Nothing else has gone wrong’ the leg is mullered anyway, we know about that, that can be dealt with. The hernia, that’s not causing me any grief. It’s just the breathing side of things.”* (P014, aged 58 yr, SPPB = 5, Stopped pulmonary rehabilitation)
Multidimensional loss	*“There is no real, I don’t know what the right word is, I want to say ‘existence.’ There is no purpose, there’s nothing. It’s wake up, if you’re lucky enough to get quality sleep. Most days it’s wake up, have a cup of tea, nebulizer, strap this thing around my nose again, sit there. There is nothing.”* (P014, aged 58 yr, SPPB = 5, Stopped pulmonary rehabilitation)
Adapting to a changing self	*“I like doing the housework and that, and I can't really do it now. I get up to do it and my back starts aching, my legs start aching, my breathing...I have to sit down, love. I do my own washing and I do my own cooking, but even when I go out and am cooking, I have to go out there and get everything ready. Then, before I put it on, I have to come and sit down.*” (P007, aged 84 yr, SPPB = 4, Completed pulmonary rehabilitation)

*Definition of abbreviations*: COPD = chronic obstructive pulmonary disease; SPPB = short physical performance battery.

**Table 3.** Tensions of balancing support with independence: illustrative quotes

Subthemes	Illustrative Quote
Filling the gaps	*“I went through one stage not so long ago where I was struggling to actually wash, as such, because of my breath.”*... *“[My wife will] wash my hair. I find this (mimes washing hair) I start and I'm trying to put my hands up, but I can sit on the chair. She'll wash all my back and that.”* ... *“I would struggle without her, no doubt about it.”* (P018, aged 64 yr, SPPB = 8, Stopped pulmonary rehabilitation)
Negotiating the right balance	*“Because every now and again I think, ‘Phone up the doctors and say, “I feel really down.’ ” And I thought, ‘Pull yourself together. No, you don’t. You’re wasting their time,’ so that’s it.”* (P015, aged 82 yr, SPPB = 3, Did not start pulmonary rehabilitation)
Mismatches and mistrust	*“So it's difficult when you're filling out forms or anything because they say, ‘Well, how far can you walk?’ and you say, ‘Well, she can walk to the gate.’ Then, the next week, she can't even get to the front door.”* (C013; P013 aged 88 yr, SPPB = 4, Stopped pulmonary rehabilitation) “*That’s what annoyed me because what I was telling them, they weren’t taking any notice and that really gets on your nerves because you’re the one in pain.*” (P004, aged 66 yr, SPPB = 5, Completed pulmonary rehabilitation)
Compounding effects of inaccessibility	*“And then when I went, it cost me a fortune. It was costing me £40 a week on cabs. I said, ‘I can’t afford this.’ Especially after Christmas. So I said, ‘I can’t afford it’.”* (P011, aged 62 yr, SPPB = 6, Stopped pulmonary rehabilitation)

*Definition of abbreviation*: SPPB = short physical performance battery.

**Table 4.** Pulmonary rehabilitation is a challenge worth facing: illustrative quotes

Subtheme	Illustrative Quote
Seeking a change	*“I’ll go and try anything, I’ve done that a lot, I thought I’ll get there somehow but do something positive. As long as I’m doing something positive to help myself, if you like, I’ll do it.”* (P004, aged 66 yr, SPPB = 5, Completed pulmonary rehabilitation)
Physically and mentally challenging	*“The other side is, at first, the strain on the body is quite severe. Well, it tends to be and psychologically it’s ‘it’s taken me two or three days to get over it. When am I going to get over it the next lot?’.”* (P003, aged 87 yr, SPPB = 6, Completed pulmonary rehabilitation)
Safe and encouraging atmosphere	*“I suppose because the safety net is it's a hospital. It's not just the physiotherapist in the hall, which it was before. Mind you one was in the hospital. But here, they're really on the ball.”* (P012, aged 74 yr, SPPB = 9, Completed pulmonary rehabilitation)
But it’s worth it	P003: *“And I definitely feel the difference.” *Interviewer: *“Yes, in what way?”* P003: *“More fluid in my movements and not so breathless, and my confidence is coming back.”* (P003, aged 87 yr, SPPB = 6, Completed pulmonary rehabilitation)

*Definition of abbreviation*: SPPB = short physical performance battery.

**Table 5.** Overcoming unpredictable disruptions to participation: illustrative quotes

Subtheme	Illustrative Quote
Determination despite disruption	*“Well, I was due to start on the 11th, and I was having a really bad breathing time, so I—so I phoned them up and said I couldn’t do it. I said, ‘I will try and get there next week.’ ”* (P017, aged 74 yr, SPPB = 9, Did not start pulmonary rehabilitation)
Rapport and flexibility of services	*“I did Wednesday and Friday. But then I couldn’t cope with Friday.”*…*“I did go, and I said, ‘I can’t do Fridays.’ ”* (P016, aged 78 yr, SPPB = 1, Stopped pulmonary rehabilitation)
No longer seen as a good fit	*“I did the first one and then later on when I’d been in hospital again they put me in for it again, but I didn’t go the second time. I’d noticed that the distance from my car to the gym was harder, so I knew if I went this time I probably wouldn’t walk that distance. I couldn’t put the car any nearer, so I thought. ‘Oh, well.’ ”* (P009, aged 82 yr, SPPB = 7, Did not start pulmonary rehabilitation)

*Definition of abbreviation*: SPPB = short physical performance battery.

### Striving to Adapt to Multidimensional Loss

Participants reported an accumulation of health events and symptoms, describing their health as “quite up-and-down” over the preceding years. Overall, they described feeling not as well as they used to be and were accustomed to facing multiple health concerns. Participants described multidimensional loss across different areas of their lives, from loss of mobility and usual activities, to loss of relationships and life-space (mobility within their community), and loss of confidence and motivation. These primary concerns were often a result of their persistent breathlessness and reduced mobility but were also influenced by low energy, pain, throat-related symptoms, decreasing memory and cognition, anxiety, and poorer strength and balance.

In the face of multidimensional loss, participants described resilience and capacity to keep adapting to a changing self. They were driven by their priorities of maintaining a sense of normality, remaining independent, and staying connected with others. This included changing how they did something (e.g., using walking aids, asking for help) and/or changing how they thought about it (e.g., accepting a slower pace, deciding something was no longer important). Whereas some adaptations were automatic and straightforward, others were emotionally challenging, particularly those involving accepting limitations. However, where losses kept building, some found it harder to keep up, and they could begin to experience a loss of purpose in their existence. This appeared more common in those living alone.

### Tensions of Balancing Support with Independence

As part of adapting, participants experienced tensions of balancing support and independence. Health and social care professionals, plus families and friends where present, often helped with filling the gaps and supporting their adaptation as it became more difficult to do things without assistance. However, this required negotiating the right balance between persevering alone and asking for help. This was an ongoing process of figuring out, by themselves and with others, how to adapt in a way that still maintained some sense of independence and did not make them feel like a burden. Against a background discourse about underresourced and overstretched services, some found this difficult.

Achieving the right balance was also made harder by instances of mismatches and mistrust. Mismatches occurred when people received conflicting advice (e.g., from specialists for different health conditions) or when services were offered routinely or reactively, rather than responding flexibly and proactively to fluctuating needs. For example, one participant described her difficulty getting a walk-in shower from local services, who presumed she did not need one because she had turned down their offer of a chairlift. She also noted the challenges of expressing her needs when her health can be so varied, for example, needing more support when she had a chest infection. Mistrust resulted from confusion or uncertainty around care, such as juggling multiple appointments, and not being clear on the purpose of the appointments. Mistrust could also stem from disagreements about appropriate support (e.g., when family were felt to overstep), and poor communication with, or not feeling listened to by, those providing support. These experiences could fracture relationships and create feelings of abandonment.

The final influence on this balancing act was the compounding effects of inaccessibility. People mentioned disabling systemic barriers to getting support to suit their needs, such as things being too costly and/or physically inaccessible. When already concerned about being a burden, feeling frustrated by mismatches, and/or having lost trust in services, people were less likely to feel accessibility issues could be overcome and would disengage.

### Pulmonary Rehabilitation Is a Challenge Worth Facing

Against this background of adapting to multidimensional loss and negotiating support and independence, participants had agreed to an assessment for pulmonary rehabilitation. All were motivated by a desire for change, whether to improve their health and symptoms or looking for an opportunity to get out of the house.

Those who attended pulmonary rehabilitation described it as physically and mentally challenging. As participants were often experiencing high levels of breathlessness and low energy, it was physically demanding to travel there and complete the exercises, and psychologically challenging to overcome their fears (e.g., of overexerting, of injuring themselves) and stay motivated. Yet at the same time, most participants, including some who stopped attending pulmonary rehabilitation part-way through their program, emphasized the benefits of experiencing a safe and encouraging atmosphere at pulmonary rehabilitation. This resulted from skilled and supportive staff, appropriate tailoring of exercises and monitoring throughout, and being in a group with people like themselves. This type of atmosphere helped to address their fears around the safety of exercising “at their age” and with their particular health experiences (e.g., heart conditions, arthritis, stroke history, recent falls).

When people then perceived benefits from participating, this also motivated ongoing engagement with pulmonary rehabilitation. People also described regaining strength, energy, control over their breath, increased motivation, and confidence—often reflecting the areas where they had previously described losses. A few participants were less sure about physical benefits, particularly when comparing with previous attendance or function. However, they often still described social and psychological benefits of participating in a group activity outside of their homes, particularly when living alone. Consequently, attendees felt participation was worth it, despite the physical and psychological demands.

### Overcoming Unpredictable Disruptions to Participation

For participants who did not start, stopped attending, or missed sessions within their pulmonary rehabilitation, the unpredictable causes of these disruptions were often apparent. Common challenges included periods of illness (e.g., exacerbation of their COPD, worsening of coexisting condition) or conflicting priorities (e.g., other areas of self-care, healthcare appointment for themselves or someone they care for, attending a funeral).

In many cases, these participants remained motivated and keen to return when they were able. Some were determined despite these disruptions: they still saw pulmonary rehabilitation as a challenge worth facing and as a good fit to support their ongoing adaptation and address their priorities. For others, it was also the rapport and flexibility of services that helped overcome disruptions. This included feeling able to discuss canceling or moving single sessions when unexpected events arose, and for two participants, negotiating less frequent attendance (i.e., once per week) when twice per week felt like too much.

However, there were circumstances when people felt unable to overcome a disruption and pulmonary rehabilitation was no longer seen as a good fit. In some cases, disruptions were simple yet crucial issues around cost or physical accessibility. For others, a deterioration in health meant they now felt it was not going to address their needs or that it was physically beyond their capabilities. This type of disruption eroded their motivation to attend, and they stopped engaging with the service.

## Discussion

People with COPD and frailty experience accumulating, multidimensional loss. In striving to adapt, participants report having to negotiate the right balance for them between independence and support. Often pulmonary rehabilitation contributed to this balance: as an opportunity to actively improve their own health, with encouragement and support from others. These positive experiences and outcomes motivated participants to attend pulmonary rehabilitation, despite its physical and mental challenges. However, owing to fluctuating health and multimorbidity, several participants experienced multiple unpredictable disruptions to completing their program. Rapport with and flexibility from service providers helped overcome these disruptions for some. For others, this disruption meant they no longer felt able to engage with pulmonary rehabilitation.

The multidimensional impact of living with COPD (39–42) is well understood. However, people’s experience of multidimensional loss may be accelerated in the context of both COPD and frailty, owing to a combination of respiratory symptom burden and low physiological reserve. The multiple challenges people experience in completing pulmonary rehabilitation (43–47) are also well established. Yet, our study demonstrates how characteristics and correlates of frailty (e.g., low energy, weakness, vulnerability to health events) can create specific challenges to participation. Considering the potential benefits of pulmonary rehabilitation for people with COPD and frailty described by our participants and in previous quantitative studies (11, 19, 20), efforts to address these challenges should be prioritized. Such initiatives may need to build reserves and resilience by better addressing multidimensional needs, working flexibly around their fluctuating health, and engendering empathetic and supportive communication around this intervention.

Interventions with potential to build resilience around multidimensional losses, and reduce the impact of unpredictable disruptions to health, may have greatest benefit for people with both COPD and frailty. Person-centered approaches suited to heterogeneity, complexity, and multimorbidity are required. One strategy potentially suited to this population might be integration with geriatric specialists to address reversible frailty causes, polypharmacy, and malnutrition (48). Comprehensive Geriatric Assessments have been effective in supporting people living with frailty in inpatient (49) and outpatient (50) settings and have been successfully used prior to surgery (51, 52) and chemotherapy (53) to improve subsequent outcomes. Exercise therapy is commonly neglected in frailty management (54), and growing evidence supports a role for inpatient geriatric rehabilitation services (55). Integrating geriatric expertise alongside outpatient pulmonary rehabilitation for people with COPD and frailty could therefore be an efficient approach. This and other interventions designed to identify and address wider support needs (e.g., better incorporation of occupational therapy (56)) could be beneficial.

Adjusting pulmonary rehabilitation services to be more flexible and responsive may also be appropriate for people with both COPD and frailty. Participants’ descriptions of balancing independent adaptation and support-seeking reflected how, as stressors on their health increase and decreased, the amount of personal and professional resource required to adapt was equally variable (31). However, professional services, including pulmonary rehabilitation, were not always responsive to their fluctuating health states. Standardized processes (e.g., discharge after a set number of missed sessions), although in some cases appropriate, were less helpful for this group.

Service adaptations that foster greater rapport with, and flexibility for, those identified as also living with frailty might facilitate participation. For example, services could offer closer supervision, preempt potential disruptions, and create shared plans for when this might occur. They could also make additional follow-up contacts before discharging people missing sessions, and/or prioritize reentry of those with frailty into services. This might be helped by more nuanced criteria for completion than the current binary approach. Instead, services could consider the number of sessions attended, note achievement of personalized goals, and acknowledge when sessions are missed owing to uncontrollable events. Such strategies to work flexibly with people with both COPD and frailty should be codeveloped to maximize acceptability, uptake, and potential effectiveness. Incorporation of home-based rehabilitation may also be helpful and can be an effective way of engaging people who cannot or prefer not to attend center-based pulmonary rehabilitation (57, 58). However, it is important to acknowledge the risk of further fragmenting support for this complex population (59), and that home-based approaches may overlook our participants’ experiences of social isolation and the benefits of a center-based group.

Finally, we must consider carefully how we communicate around pulmonary rehabilitation. As people with both COPD and frailty experience more unpredictability, they may increasingly use emotion-focused adaptations. Emotion-focused adaptations may include reframing perceptions of themselves and/or pulmonary rehabilitation such that it is not seen as a good fit (e.g., “I’m not well enough to participate, the service is too much for me”) (30). To avoid disengagement, those working with people with COPD and frailty may need to communicate in a way that helps realign their perceptions of themselves and pulmonary rehabilitation.

Helpful approaches might include empathetic communication suggesting problem-focused strategies to support engagement (e.g., offering walking aids, focusing on falls within rehabilitation), but also importantly addressing the emotional aspects of their experience (e.g., building confidence, working through fears and misconceptions, emphasizing the social and safe environment, linking rehabilitation to their priorities). This could include learning from the success of motivational interviewing-based health coaching (60). If pulmonary rehabilitation is truly no longer the best fit for their goals, these conversations might also provide opportunities to discuss other suitable interventions. For example, lower-intensity exercise-based services (e.g., chair exercise, yoga (61)), breathlessness services that integrate palliative care expertise (62), and/or community groups with more social emphasis (e.g., singing groups (63)) may suit that individual. However, with evidence of the benefits of pulmonary rehabilitation for people with both COPD and frailty, prioritizing this as a first-line approach remains important.

Purposive sampling facilitated inclusion of diverse experiences, including those who stopped attending, and those who never commenced, pulmonary rehabilitation. However, our sample was limited to two urban sites, mainly to people with white ethnicities, and to people who attended their initial assessment for pulmonary rehabilitation. Approximately one in three people referred to pulmonary rehabilitation in the United Kingdom do not attend their initial assessment (64). This limits the theoretical transferability of our findings. Social desirability bias may have influenced honesty about services, and the presence of family members during some interviews may have affected responses. For some, however, family members seemed to facilitate recall of events and reporting of needs. The analysis being led by an individual with psychology training may have increased the focus on psychosocial concerns; therefore, involvement of others at different stages of the analysis was used to mitigate against this. Given recent debate regarding using the concept of “saturation” within reflexive thematic analysis (65), we have made efforts to be transparent about how this was operationalized within our study. This study used relevant existing theory and the input of service user representatives to inform the data collection, analysis, and interpretation to strengthen credibility and interpretive rigor.

### Conclusions

People living with both COPD and frailty experience accumulating, multidimensional loss. This group are motivated to complete pulmonary rehabilitation but often require additional support and flexible services owing to their unpredictable health. Person-centered approaches suited to people with multiple conditions should be considered to minimize disruptive health events and support pulmonary rehabilitation attendance. Alongside this, services need to prioritize supportive communication around how pulmonary rehabilitation may align with participant’s priorities and consider more flexible delivery models to meet the fluctuating needs of this group.

## Supplementary Material

Supplements

Author disclosures
