# Supplementary material for: Experiences of Pulmonary Rehabilitation in People Living with Chronic Obstructive Pulmonary Disease and Frailty. A Qualitative Interview Study
Source: Ann Am Thorac Soc. 2020 Oct;17(10):1213–21. doi: 10.1513/AnnalsATS.201910-800OC (PMC7640624; doi:10.1513/AnnalsATS.201910-800OC)
Supplement: Supplements [file AnnalsATS.201910-800OC.html]

Experiences of Pulmonary Rehabilitation in People Living with Chronic Obstructive Pulmonary Disease and Frailty. A Qualitative Interview Study | Annals of the American Thoracic Society

- brighton\_data\_supplement.pdf (232 KB)
- disclosures.pdf (220 KB)
