# Supplementary material for: Experiences of Pulmonary Rehabilitation in People Living with Chronic Obstructive Pulmonary Disease and Frailty. A Qualitative Interview Study
Source: Ann Am Thorac Soc. 2020 Oct;17(10):1213–21. doi: 10.1513/AnnalsATS.201910-800OC (PMC7640624; doi:10.1513/AnnalsATS.201910-800OC)
Supplement: Supplements [file AnnalsATS.201910-800OC_brighton_data_supplement.pdf]

Online supplementary material

Brighton LJ, Bristowe K, Bayly J, Ogden M, Farquhar M, Evans CJ, Man WD, Maddocks M. Experiences of pulmonary rehabilitation in people living with COPD and frailty: a qualitative interview study. Annals of the American Thoracic Society. 2020. DOI: 10.1513/AnnalsATS.201910-800OC

Supplementary material E1: Interview topic guide

Current health and function:	
1) It is helpful for us to know about how you've been getting on day to day. Can you please tell me about your health over the last couple of months?	How are you getting on now?<br>Do you think this will change?<br>How much does this impact on the things you like to do?<br>Is this something you think can be changed?<br>Are there any concerns you have about your health?
2) Can you please tell me, when thinking about your health, what's most important to you?	How do you try to achieve these at the moment?<br>What motivates you to do this?<br>How do you know if something works?
**Which services and professionals people are currently accessing:**	
3) Who currently supports you in relation to your health?	In what way?<br>Family, Friends, others in the community?<br>GP, other doctors, nurses, other professionals?
**How their concerns are being addressed:**	
4) Is there anything that is worrying you about your health that is not being addressed at the moment?	This could be about your physical health,<br>how you feel in yourself, relationships,<br>or other aspects of health and wellbeing.
**Their expectations of pulmonary rehabilitation:**	
5) Please can you tell me about what you expected the pulmonary rehabilitation service to be like?	What did you think you might get out of it?<br>How did you think it would help with that?<br>What concerns did you have about participating?<br>Did you think it would help in the long-term?
**Their experience of pulmonary rehabilitation:**	
6) Could you please talk me through what it was like for you when you then attended the pulmonary rehabilitation service?	Completer prompts:<br>How did it differ to what you expected?<br>What did you get out of it, if anything?<br>How could you tell?<br>How do you think it helped with that?<br>Were there any challenges or downsides?<br>How do you think it could be improved?
	Stopped attending prompts:<br>How did it differ to what you expected?<br>What did you get out of it, if anything?<br>How could you tell?<br>How do you think it helped with that?<br>Were there any challenges or downsides?<br>How do you think it could be improved?<br>What were your reasons for not going back?
	Did not attend prompts:<br>What were your reasons for not going to the sessions?
7) Is there something else you would like to tell me before we finish?	Thank you.

Supplementary material E2: Consolidated criteria for reporting qualitative research (COREQ) checklist

**No. Item**	**Guide questions/description**	**Reported on Page #**
**Domain 1: Research team and reflexivity**		
Personal Characteristics		
1. Inter viewer/facilitator	Which author/s conducted the interview or focus group?	3
2. Credentials	What were the researcher's credentials? E.g. PhD, MD	3
3. Occupation	What was their occupation at the time of the study?	3
4. Gender	Was the researcher male or female?	3
5. Experience and training	What experience or training did the researcher have?	3
Relationship with participants		
6. Relationship established	Was a relationship established prior to study commencement?	3
7. Participant knowledge of the interviewer	What did the participants know about the researcher? e.g. personal goals, reasons for doing the research	3
8. Interviewer characteristics	What characteristics were reported about the inter viewer/facilitator? e.g. Bias, assumptions, reasons and interests in the research topic	3
**Domain 2: study design**		
Theoretical framework		
9. Methodological orientation and Theory	What methodological orientation was stated to underpin the study? e.g. grounded theory, discourse analysis, ethnography, phenomenology, content analysis	2
Participant selection		
10. Sampling	How were participants selected? e.g. purposive, convenience, consecutive, snowball	2-3
11. Method of approach	How were participants approached? e.g. face-to-face, telephone, mail, email	2-3
12. Sample size	How many participants were in the study?	3
13. Non-participation	How many people refused to participate or dropped out? Reasons?	3-4
Setting		
14. Setting of data collection	Where was the data collected? e.g. home, clinic, workplace	3
15. Presence of non-participants	Was anyone else present besides the participants and researchers?	3
16. Description of sample	What are the important characteristics of the sample? e.g. demographic data, date	3, Table 1
Data collection		
17. Interview guide	Were questions, prompts, guides provided by the authors? Was it pilot tested?	3, Supplement E1
18. Repeat interviews	Were repeat inter views carried out? If yes,	No

	how many?	
19. Audio/visual recording	Did the research use audio or visual recording to collect the data?	3
20. Field notes	Were field notes made during and/or after the inter view or focus group?	3
21. Duration	What was the duration of the inter views or focus group?	4
22. Data saturation	Was data saturation discussed?	3, 7
23. Transcripts returned	Were transcripts returned to participants for comment and/or correction?	No
**Domain 3: analysis and findings**		
Data analysis		
24. Number of data coders	How many data coders coded the data?	3
25. Description of the coding tree	Did authors provide a description of the coding tree?	Tables 2-5
26. Derivation of themes	Were themes identified in advance or derived from the data?	3
27. Software	What software, if applicable, was used to manage the data?	NVivo 12
28. Participant checking	Did participants provide feedback on the findings?	No
Reporting		
29. Quotations presented	Were participant quotations presented to illustrate the themes/findings? Was each quotation identified? e.g. participant number	Tables 2-5
30. Data and findings consistent	Was there consistency between the data presented and the findings?	4-5, Tables 2-5
31. Clarity of major themes	Were major themes clearly presented in the findings?	4-5, Tables 2-5
32. Clarity of minor themes	Is there a description of diverse cases or discussion of minor themes?	4-5, Tables 2-5
